# Effect of Mass Transport on the Electrochemical Oxidation of Alcohols Over Electrodeposited Film and Carbon-Supported Pt Electrodes

**DOI:** 10.1007/s11244-018-0893-6

**Published:** 2018-01-19

**Authors:** Vinod Kumar Puthiyapura, Wen-Feng Lin, Andrea E. Russell, Dan J. L. Brett, Christopher Hardacre

**Affiliations:** 1 School of Chemical Engineering and Analytical Science, The University of Manchester, Manchester, M13 9PL UK; 2 Department of Chemical Engineering, Loughborough University, Loughborough, Leicestershire LE1 13TU UK; 3 Department of Chemistry, University of Southampton, High Field, Southampton, SO17 1BJ UK; 4 Department of Chemical Engineering, University College London (UCL), London, WC1E 7JE UK

**Keywords:** Direct alcohol fuel cells, Rotating disk electrode (RDE), Electro-oxidation, Methanol, Ethanol, Butanol, Platinum

## Abstract

Electrochemical oxidation of four different alcohol molecules (methanol, ethanol, *n*-butanol and 2-butanol) at electrodeposited Pt film and carbon-supported Pt catalyst film electrodes, as well as the effect of mass transport on the oxidation reaction, has been studied systematically using the rotating disk electrode (RDE) technique. It was shown that oxidation current decreased with an increase in the rotation rate (ω) for all alcohols studied over electrodeposited Pt film electrodes. In contrast, the oxidation current was found to increase with an increase in the ω for Pt/C in ethanol and *n*-butanol-containing solutions. The decrease was found to be nearly reversible for ethanol and *n*-butanol at the electrodeposited Pt film electrode ruling out the possibility of intermediate CO_ads_ poisoning being the sole cause of the decrease and was attributed to the formation of soluble intermediate species which diffuse away from the electrode at higher ω. In contrast, an increase in the current with an increase in ω for the carbon supported catalyst may suggest that the increase in residence time of the soluble species within the catalyst layer, results in further oxidation of these species. Furthermore, the reversibility of the peak current on decreasing the ω could indicate that the surface state has not significantly changed due to the sluggish reaction kinetics of ethanol and *n*-butanol.

**Electronic supplementary material:**

The online version of this article (10.1007/s11244-018-0893-6) contains supplementary material, which is available to authorized users.

## Introduction

**Scheme 1:**
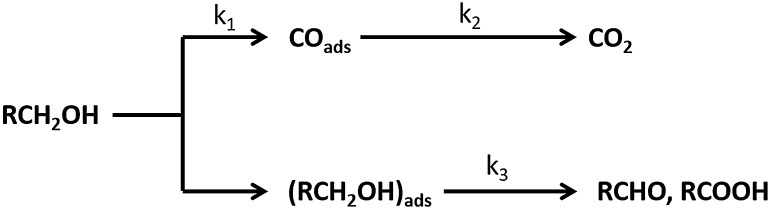
The dual path mechanism of alcohol oxidation reaction [25]

The electrochemical oxidation of alcohols is an important reaction in direct alcohol fuel cells (DAFCs). Due to their relatively high reactivity for electro-oxidation, methanol and ethanol have been widely studied as a fuel for DAFCs [1–3]. Recently, second-generation biofuels such as butanol have also attracted attention as alternative fuels to methanol and ethanol for the DAFCs [4, 5]. Pt is known as the most active single metal catalyst for alcohol oxidation reaction in acidic media and has been studied extensively for methanol [6–10], ethanol [11–13] and butanol isomers [4, 5, 14–16]. The intermediates formed during the oxidation of the alcohol molecules on the Pt surfaces have been identified using various electrochemical and spectroscopic techniques such as in-situ FT-IR [17, 18] and *online* differential electrochemical mass spectroscopy (DEMS) [18–23]. Based on these studies, a dual path reaction mechanism (Scheme 1) has been proposed and well documented for small chain alcohol molecules [24].

Alcohol molecules have two active sites (OH group and α carbon atom) that can interact with the metal catalyst during the adsorption process [26] and they can adsorb onto the catalyst surface through either of these sites by breaking the O–H or C–H bond to form adsorbed intermediates before the cleavage of C–C and/or C–O bonds, as given in Eqs. (1) and (2) [26, 27]. This is considered as the first step in the oxidation of alcohols [28].12

Both C- and O-bound adsorbed species could have a proton removed and form an aldehyde (–CHO) species. According to the dual path mechanism for primary alcohols (Scheme 1) the reaction follows two parallel pathways: (i) adsorption of alcohol to the catalyst surface and direct dehydrogenation to form CO_ads_ which may further oxidise to form CO_2_ and (ii) a series of non-adsorbing intermediate species forming partial oxidation products [29, 30]. Due to the complex reaction pathways and formation of various adsorbed intermediates, alcohol oxidation reaction is generally sluggish on Pt. With the increase in carbon chain length, the electro-oxidation reactivity of alcohols was observed to decrease on Pt as in the order methanol > ethanol > propanol > butanol [31, 32] due to the difficulty in C–C bond cleavage. The high overpotential requirement for the alcohol oxidation reaction and the deactivation due to CO_ads_ poisoning are still the major obstacles for the alcohol oxidation reaction [33, 34]. The removal of CO_ads_ from the Pt surface requires an oxidant, for example, OH_ads_, which is formed by the oxidation of water only at relatively high potentials [35].

For the methanol oxidation reaction (MOR), CO_2_ is formed together with significant quantities of HCHO and HCOOH on Pt [21, 29, 30, 36, 37]. A CO_2_ generation current efficiency less than 100% indicates a parallel reaction pathway towards partial oxidation products [7, 21, 36, 38]. A DEMS study for the ethanol oxidation reaction (EOR) on Pt has shown that EOR mainly forms CO_2_, CH_3_CHO and CH_3_COOH [18, 22, 39, 40]. Mostafa et al. [22] observed a CO_2_ product yield of 1% in comparison to CH_3_CHO (55%) and CH_3_COOH (44%) during EOR on Pt/C. Ethane 1,1 diol [11] was also detected as a product of EOR which forms as result of hydrolysis of CH_3_CHO.

The electrochemical activity of the electrocatalysts is usually studied under steady-state conditions in a three-electrode system. However, in a real fuel cell, the fuel is generally fed into the anode of DAFCs; i.e. the system is under non-steady state condition. It is thus important to understand the effect of fuel transport to the catalyst on its activity. The maximum oxidation current is generally limited by the formation of Pt oxides and not the diffusion of the alcohol [41, 42]. Thus, the alcohol oxidation current is generally not expected to increase with an increase in mass transport. Since water is present in large excess, the reaction of water to give OH_ads_ is also not considered to be influenced significantly by mass transport [38]. An increased flow rate of alcohol is usually found to decrease the fuel cell membrane electrode assembly (MEA) activity and this was mainly attributed to the alcohol crossover through the proton exchange membrane to the cathode side [43, 44]. However, moderate flow rates also help in the removal of the product CO_2_ generated in the flow field. A slow flow rate, on the other hand, may result in depletion of the alcohol, especially at high current density [45], reducing the MEA performance. Not many reports dedicated to studying the effect of flow rate on the catalyst activity have been documented in the literature. Katayangi and Yamazaki [46] observed a decrease in CO_2_ production in a single ethanol fuel cell on increasing the ethanol flow rate with Pt/C anode catalyst and attributed this to the dominant formation of acetaldehyde.

A few half-cell studies are available on the effect of mass transport upon alcohol oxidation over Pt, but these have concentrated on the utilisation of methanol as a fuel [38, 41, 42, 47–49] with very few studies using ethanol [39, 50, 51]. Most studies have reported a suppression of the extent of oxidation of methanol on Pt disk electrode with increased mass transport [38, 42]. This has been attributed to the enhanced production of soluble species which diffuse away from the catalyst surface at the expense of CO_2_ production [38]. However, not all studies are in agreement. For example, Hou et al. [52] reported an increase in peak current for methanol oxidation current on increased rotation rate (ω) for smooth Pt in 1 M methanol + 0.5 M H_2_SO_4_ solution. These discrepancies could be due to the different experimental conditions employed, such as scan rate, concentration, temperature, catalyst loading, electrode surface structure etc. In contrast, on carbon supported Pt, a less significant change [42] or an increase in the current [51] with an increase in ω was reported. However, methanol oxidation activity is not always a good model for long chain alcohol oxidation as it does not require C–C bond breaking.

In the study reported herein, the effects of mass transport on the electro-oxidation of higher alcohol molecules such as *n*-butanol and 2-butanol have been investigated and compared with those for methanol and ethanol using both electrodeposited Pt film and carbon supported Pt nanoparticle electrocatalysts. Also, the influence of catalyst layer thickness on the activity of Pt under increased mass transport is described in details.

## Experimental

**Fig. 1:**
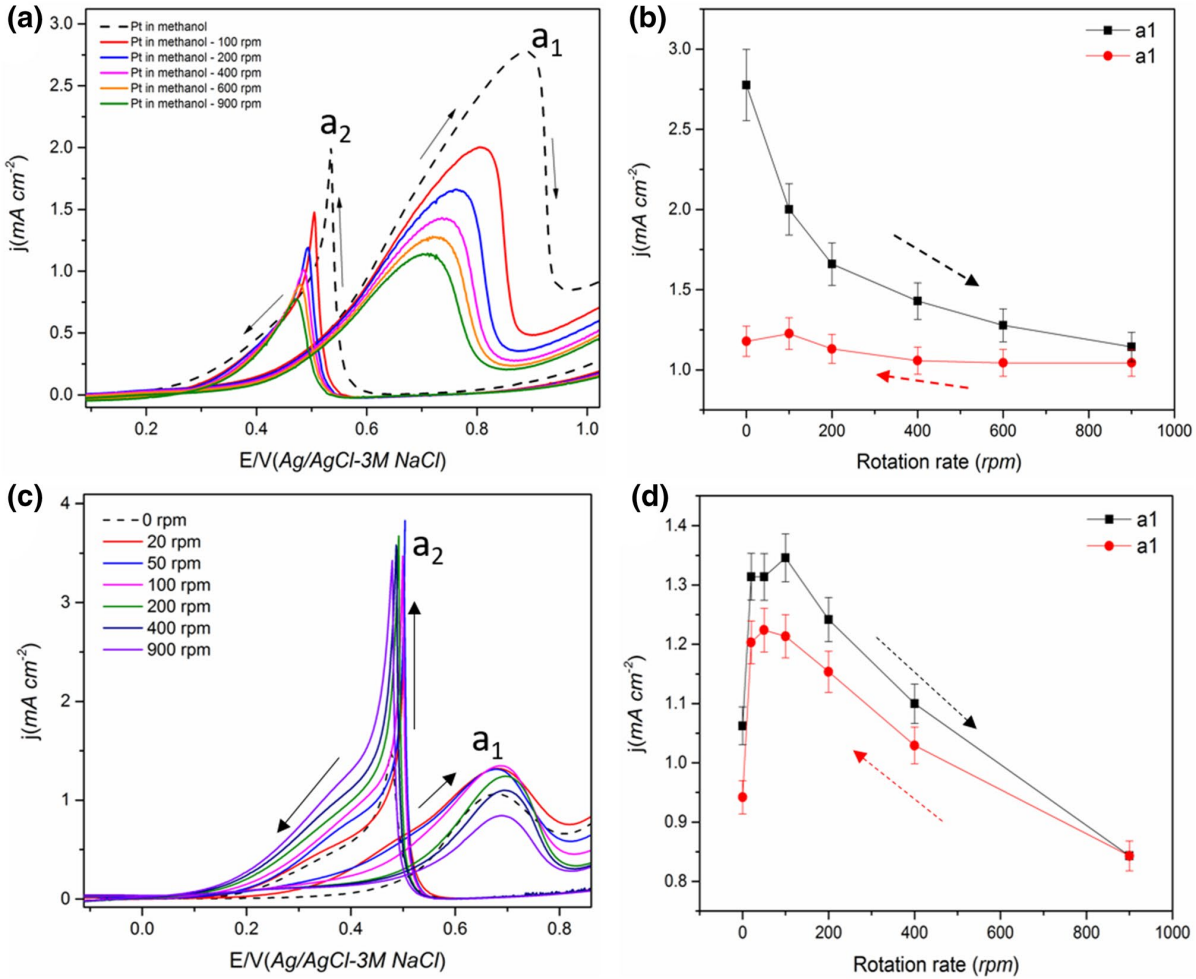
The effect of electrode rotation rate (ω) on the cyclic voltammogram of electrodeposited Pt film electrode-1 in (**a, b**) 0.5 M methanol + 0.1 M HClO_4_ and peak a_1_ current density (**c, d**) electrode-2 in 0.5 M ethanol + 0.1 M HClO_4_ and peak a_1_ current density. Scan rate 50 mVs^−1^. Solid arrow shows the direction of the potential scan

**Fig. 2:**
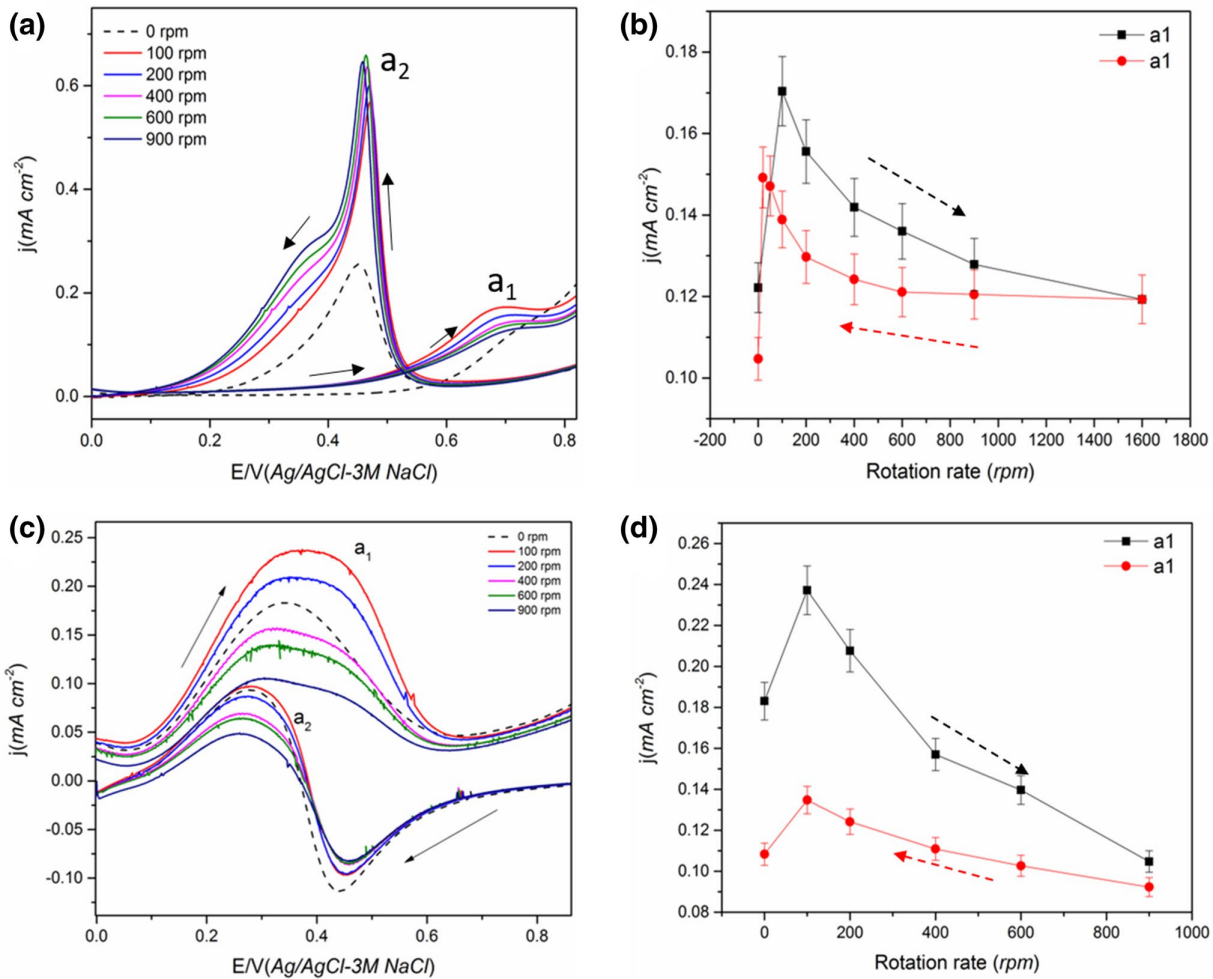
The effect of electrode rotation on the CV of electrodeposited Pt film in (**a, b**) electrode-2 in 0.5 M *n*-butanol + 0.1 M HClO_4_ solution and peak a_1_ current density. **c, d** Electrode-3 in 0.1 M 2-butanol + 0.1 M HClO_4_ solution and peak a_1_ current density. Scan rate 50 mVs^−1^ Arrow shows the direction of the potential scan. Scan rate 50 mVs^−1^

The electrochemical measurements were carried out in a standard three-electrode cell using a potentiostat (SP240, Bio-Logic) and Pine rotating disk electrode set up with a glassy carbon (GC) electrode of 5 mm diameter. A Ag/AgCl–3 M NaCl (Basi, USA) (0.210 V vs. NHE) and a Pt mesh (Goodfellow, UK) were used as reference and counter electrode respectively. All potentials are referenced with respect to Ag/AgCl–3 M NaCl unless specified otherwise. N_2_ gas was purged through the electrolyte solution for 15–20 min before carrying out the electrochemical analysis. The GC electrode was polished with 1, 0.3 and 0.05 μm alumina powder with subsequent ultrasonication in de-ionised water before use. To prepare the electrodeposited thin film electrode, Pt was electrodeposited onto the GC electrode from 5 mM H_2_PtCl_6_ + 0.1 M H_2_SO_4_ solution at a potential of − 0.22 V for 15 min (2 mM H_2_PtCl_6_ + 0.05 M H_2_SO_4_ for 30 min in Figs. 1c, 2a). The loading and the thickness of the catalyst layer for electrodeposited Pt electrode is calculated using (3) and (4), where *Q* is the charge passed during the electrodeposition assuming 4e^−^ reduction of Pt^4+^ to Pt(0), M is the molecular weight of the Pt (195.08 gmol^−1^), *n* is the number of electrons required for the deposition of one mole of Pt, *F* is the Faraday constant (96,485 C mol^−1^), *ρ* is the density of Pt (21.45 g cm^−3^) and *A* is the geometric area of the glassy carbon electrode (0.1963 cm^2^) (Table 1). The morphology of the electrodeposited Pt has been described previously [5] where a ‘cauliflower-like’ spherical morphology was observed.

**Table 1:** The Pt metal loading and layer thickness of the various electrodes used

Catalyst sample	Loading (mg cm^−2^)	Catalyst layer thickness (µm)	Notes
Electrode-1	0.48 ± 0.10	23 ± 4	Figure 1a (methanol)
Electrode-2	0.46 ± 0.30	21 ± 3	Figure 1c (ethanol) and Fig. 2a (*n*-butanol)
Electrode-3	0.38 ± 0.10	18 ± 3	Figure 2c (2-butanol)
Pt/C catalyst	0.07 (Pt)	–	Figures 4, 5
Pt/C thicker catalyst layer	0.14 (Pt)	–	Figure 6

The electrode was activated in N_2_ saturated 0.1 M HClO_4_ solution at a potential range of − 0.22 to 1.3 V for about 25–30 cycles until a stable cyclic voltammogram (CV) is obtained before being tested in alcohol containing the solution. A typical CV of Pt obtained in the acidic solution is given in Fig. S1. The electrochemical active area (A_r_) was calculated from the area of the hydrogen desorption region, which was then used to normalise the current to obtain current density [4]. A scan rate of 50 mVs^−1^ was used for all the CV tests.

3 $$m=\frac{{QM}}{{nF}}$$

4 $$Layer~thickness=\frac{{QM}}{{4F\rho A}}$$

Commercial HisPEC-4000 Pt/C (40 wt%) was used for Pt/C catalyst ink preparation. The ink was prepared by mixing 5 mg of the catalyst with 1.5 cm^3^ (ethanol + water) solvent and 20 μL Nafion solution (5 wt% solution). The latter served both as adhesive and proton conductor. The mixture was then ultrasonicated for 30 min and 10 μL ink was drop cast onto a GC electrode and dried at ambient temperature. The catalyst loading was ca.68 µg cm^−2^ (Table 1). Cyclic voltammetry was conducted in the acid supporting electrolyte prior to the collection of data in the alcohol-containing solution.

XRD of the Pt/C sample was carried out using Panalytical X-pert-Pro diffractometer with Cu Kα radiation, step size 2θ = 0.001° and a X’Celerator RTMS detector. The crystallite size and lattice constant of the Pt particles were calculated using Eqs. (5) and (6) [53] where *d* is the crystallite size, *λ* is the X-ray wavelength, *β* is the full length at half maximum, *a* is the lattice constant and *θ* is the XRD peak angle.

5 $$d=\frac{{0.9\lambda }}{{\beta \cos \theta }}$$

6 $${a_{fcc}}=\frac{{\sqrt {{h^2}+{k^2}+{l^2}} \lambda }}{{2\sin \theta }}$$

## Results and Discussion

### Methanol and Ethanol Oxidation Reaction on Electrodeposited Pt Film

The CV of Pt in methanol and ethanol containing solution showed the characteristic oxidation peaks a_1_ and a_2_ during the anodic and cathodic scan respectively as given in Fig. 1 [25]. The effect of electrode rotation on the peak a_1_ current density is also given in Fig. 1.

**Scheme 2:**
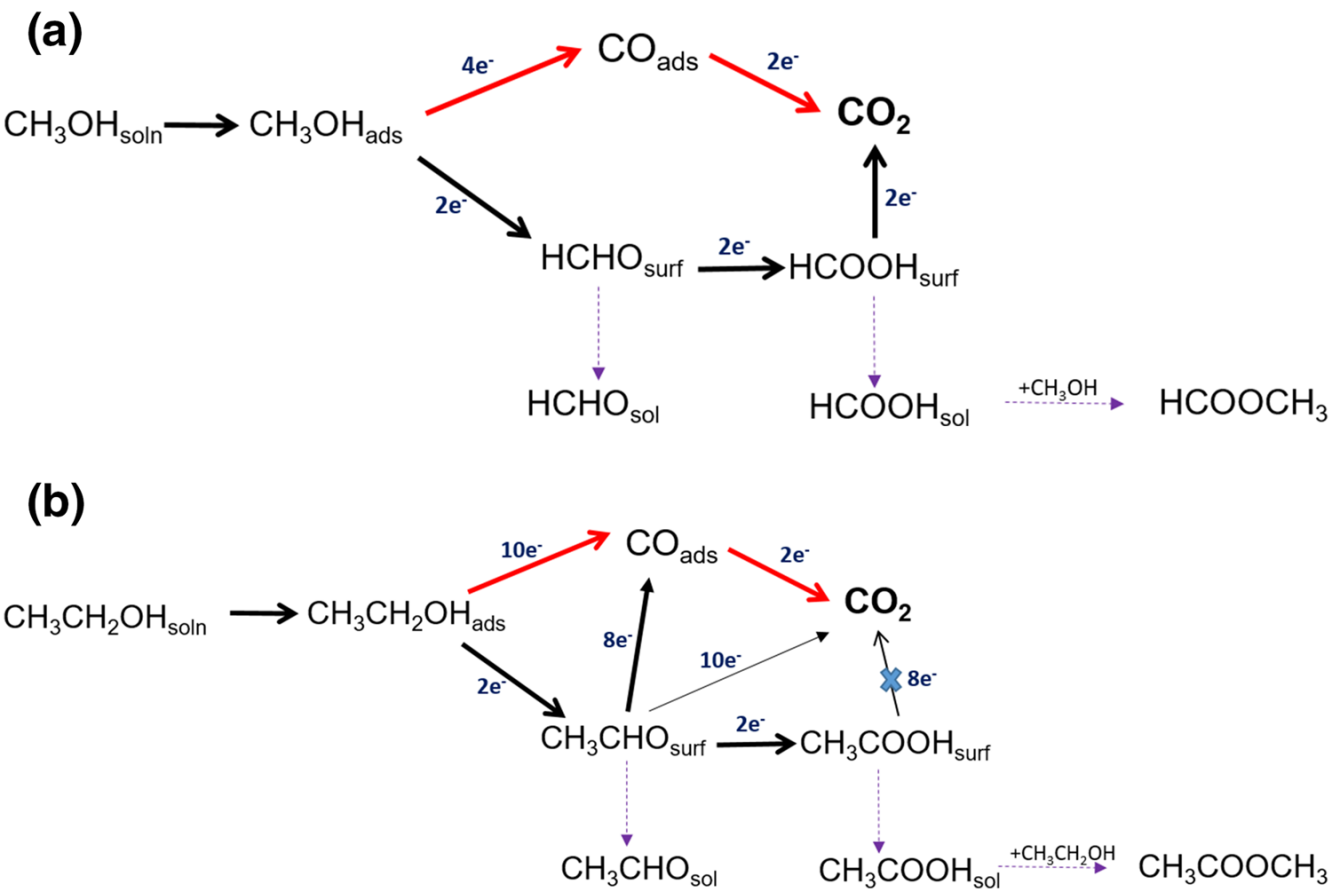
Schematic of electrochemical oxidation on Pt in acidic media of (**a**) methanol (**b**) ethanol

It is known that CO_2_ is formed together with significant quantities HCHO and HCOOH for the MOR on Pt [18, 20, 21, 23, 36, 37]. The EOR mechanism is also similar to MOR in that, it follows a dual path mechanism (Scheme 2b) [54]. However, it is much more complicated than MOR, due to the presence of the C–C bond which requires higher energy to break. Ethanol mainly undergoes partial oxidation at lower potentials to form various soluble products such as CH_3_CHO and CH_3_COOH together with small amounts of CO_2_ [11, 18, 22, 26, 54–56]. The soluble intermediate products formed can either be further oxidised to form CO_2_ at the catalyst surface or can dissolve in the electrolyte media (Scheme 2). The total current generated in the reaction can be expressed simply as Eq. (7), taking methanol as an example. The partial oxidation of the alcohol reduces the number of electrons generated per alcohol molecule and thus reduces the fuel efficiency [20, 23, 47].

7 $${I_{total}}=6~{I_{{\text{C}}{{\text{O}}_2}}}+4~{I_{{\text{HCOOH}}}}+2~{I_{{\text{HCHO}}}}$$

From Fig. 1, it is observed that the peak a_1_ current decreases with an increase in ω for both methanol and ethanol. Since peak a_1_ is associated with the corresponding alcohol oxidation, a decrease in current indicates a reduced reaction rate and/or reaction yield (number of electrons generated) with an increase in mass transport. A similar decrease in peak a_1_ current for MOR with an increase in the ω was also reported previously for smooth Pt electrode [42] and Pt_3_Co bulk alloy [41]. Considering a typical mass transport controlled reaction, the current is expected to increase with an increase in ω. The decrease indicates that the reaction is not mass transport controlled and suggests increased diffusion of reactive species away from the electrode surface with an increase in ω [38, 39, 52]. During the alcohol oxidation, a thin layer of increased concentration of CO_2_ and other intermediates compared to the bulk concentration will be formed close to the catalyst surface; on rotation, the alcohol from the bulk will have to diffuse through this thin layer where counter diffusion of CO_2_ and other soluble intermediates from the catalyst takes place [52]. i.e. the diffusion of intermediates from and convection of alcohol towards the catalyst are competing with the oxidation of the intermediates [20]. Hou et al. [52] observed a significantly lower diffusion coefficient of methanol (~ 10^−12^ cm^2^ s^−1^) compared to the typical value (~ 10^−5^ cm^2^ s^−1^) for smooth Pt in chronoamperometry study, confirming the counter diffusion of soluble species from the catalyst. The convective removal of the partial oxidation products on rotation makes them unavailable for further oxidation reducing the total number of electrons released per molecule in the reaction as given in Eq. (7) for MOR. i.e. CO_2_ is formed only by the CO_ads_ pathway at higher ω for MOR whereas, in static solution, both pathways contribute towards the current.

However, the removal of partial oxidation products and the increased consumption of alcohol should increase the formation of the soluble products (eg: CH_3_OH→HCHO for MOR) and increase the current through this pathway. Seland et al. [38] proposed that the increase in current through this pathway is surpassed by a decrease in current through the CO_ads_ pathway due to an increased CO_ads_ coverage at higher ω. They confirmed this by observing an increase in peak current following a transient increase in ω for MOR (when rotation suddenly changed from low to very high) [38, 39]. This increase was attributed to the fact that, a transient increase in ω does not affect the CO_ads_ pathway and does not change the Pt surface state but enhances the HCHO pathway and subsequently increases the total current [38, 39]. A gradual increase in ω, on the other hand, increases the adsorption of alcohol over water favouring the formation of CO_ads_ over the OH_ads_, which in turn increases the CO_ads_ coverage on the Pt surface, blocking the active sites leading to a reduced current [39, 42]. A decrease in CO_2_ current efficiency during MOR with an increase in flow rate has been observed in previous DEMS studies [20, 36, 48], confirming incomplete oxidation of methanol to CO_2_. This reasoning can also be applied to EOR (Fig. 1c and Scheme 2b).

On stopping the rotation at 900 rpm following the increase in ω, both the peak current for a_1_ and a_2_ increase slightly and on applying the rotation again, the current decreases for both methanol and ethanol (not shown). This confirms the role of the diffusion of soluble intermediates and rules out the possibility of a contaminant in the electrolyte being responsible for the decrease in peak current (increased diffusion of contaminants to the electrode) [42]. In addition, on reducing the ω, the peak a_1_ current increases for methanol and ethanol with the change found to be reversible in the case of ethanol (Fig. 1d) compared with methanol (Fig. 1b). This indicates that the surface state might have changed significantly during the increase in ω for MOR making it less active. As mentioned above, this could be due to the high coverage of CO_ads_ on the Pt surface with an increase in rotation leading to a more irreversible blocking effect. Even though a higher potential is expected to oxidise CO_ads_ completely, this will only happen if there are sufficient OH_ads_ present on the catalyst surface. The OH_ads_ coverage is likely to be reduced at higher rotation rates due to increased coverage by methanol. This indicates that the CO_ads_ or other strongly adsorbed intermediate coverage is higher at higher ω for MOR [42] decreasing the number of Pt active sites available for methanol oxidation during the subsequent decrease in ω.

The reversibility of the peak a_1_ current on decreasing the ω for EOR indicates that CO_ads_ cannot be the cause of the decrease in current. Unlike MOR, the EOR mainly leads to C_2_ intermediates [22], and the formation of strongly adsorbed intermediates such as CO_ads_ is lower than that for MOR due to the poor reaction kinetics of ethanol/lower activity for C–C bond cleavage. Therefore, in this case, the surface state of Pt is unlikely to have changed significantly with an increase in ω, as the intermediate soluble species are removed at higher ω. On decreasing the ω, the Pt active sites will then be available for adsorption and oxidation of the ethanol. Since CH_3_COOH is difficult to further oxidise at the potential range of peak a_1_ [32], the diffusing species is possibly CH_3_CHO. Also, the production of aldehyde is not significantly influenced by water whereas the production of acidic species requires water on the catalyst surface [11]. Thus, the influence of rotation on water oxidation reaction will also influence the alcohol oxidation product distribution. As the water adsorption is less dominant at higher ω, the major product and diffusing species at peak a_1_ are likely to be CH_3_CHO in the case of EOR.

The trends found for the current of peak a_2_ were the same as that of peak a_1_ for methanol, i.e. decrease with increase in ω (Fig. S2) [38, 42]. For methanol, the peak a_2_ is generally attributed to the re-oxidation of intermediates formed (eg: CH_x_O) during the anodic scan as well as fresh methanol adsorption on the oxide-free Pt surface [25, 49]. However, using in-situ surface enhanced IR absorption spectroscopy (SEIRAS), Duffy et al. [57] observed that the a_2_ peak solely comes from freshly adsorbed methanol and not from the remaining carbonaceous species from the anodic scan. The decrease in peak current could be explained as in the case of peak a_1_; i.e. diffusion of soluble intermediate species at higher ω for MOR. For EOR, unlike the trend found for MOR, the current for peak a_2_ showed an initial increase on increasing ω to 100 rpm and thereafter it remained constant with further increases in ω (Fig. S2b). The peak a_2_ for EOR associated with the oxidation of freshly adsorbed ethanol on the reduced oxide-free sites mainly forming CH_3_CHO and CH_3_COOH [18, 22, 39, 40]. No CO_2_ formation was reported for the peak a_2_ for EOR. An increase in current for peak a_2_ on rotation was also observed by Seland et al. [39]. This was thought to be due to a slow formation of adsorbed species at the potential of the peak a_2_ for ethanol compared to the fast formation of CO_ads_ for methanol. On decreasing the ω, the peak current behaved similarly to that during the increase in ω (Fig. S2b). The lack of any changes in the peak current is consistent with the fact that the reaction is not mass transport controlled.

### Butanol Oxidation on Electrodeposited Pt Film

**Scheme 3:**
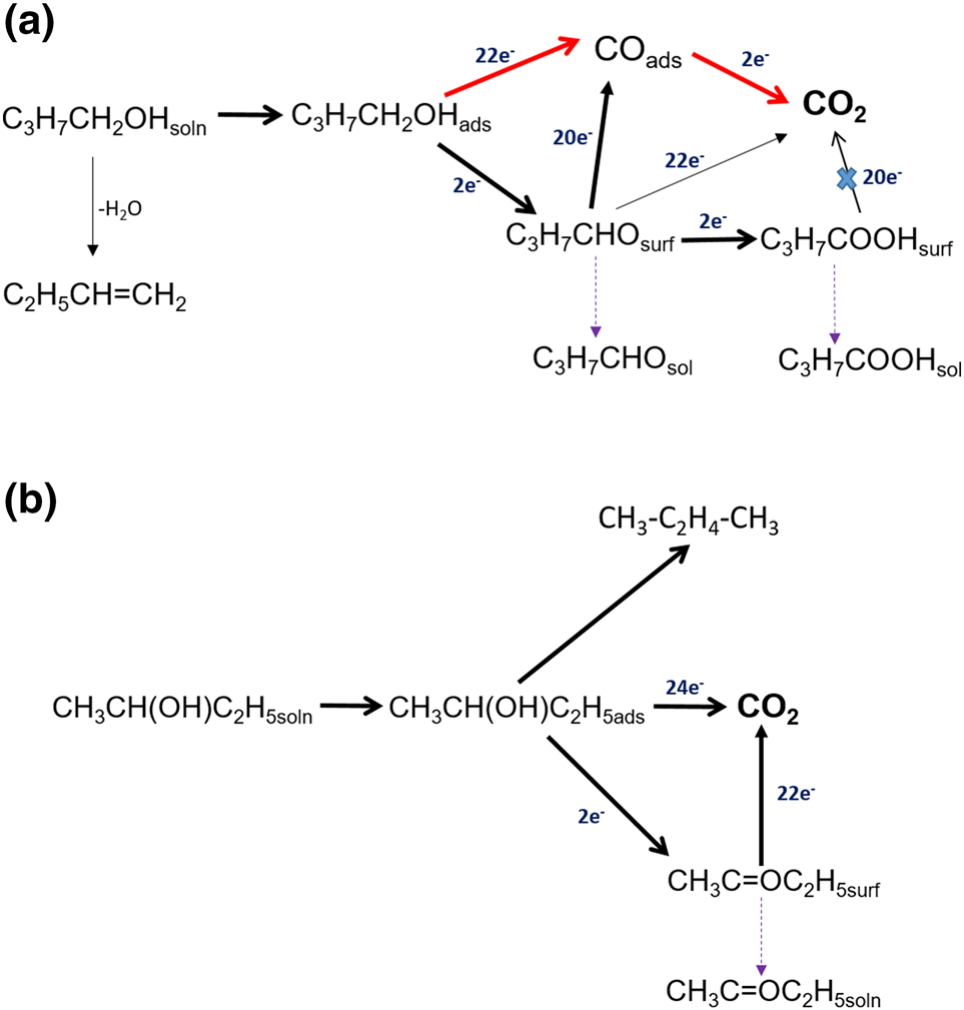
The schematic of electro-oxidation on Pt in acidic media of (**a**) *n*-butanol (**b**) 2-butanol

As found with methanol and ethanol, the current density for peak a_1_ decreased with an increase in ω for both *n*-butanol and 2-butanol as shown in Fig. 2. The near reversibility of peak a_1_ current on decreasing ω was also observed for *n*-butanol as in the case of ethanol; whereas the 2-butanol behaved similarly to methanol with poor reversibility (Fig. 2d). The same reasoning for ethanol can be applied for *n*-butanol, although it should be noted that the CO_ads_ coverage will be even lower in the case of *n*-butanol than during EOR. Mukherjee and Bhattacharya [58] observed the formation of aldehyde and ester groups during *n*-butanol oxidation on Pt and Pd electrodes in alkaline media using in-situ FTIR and this was attributed to butyraldehyde and butyric acid formation. Nan-Hai and Shi-Gang [33] also conducted an in-situ FTIR study of *n*-butanol on Pt in acidic media and concluded that the formation of butyric acid (or via butyraldehyde) is the major reaction pathway, indicating that the complete oxidation to CO_2_ is low (Scheme 3). Gootzen et al. [59] observed the formation of CO_2_ in the peak a_1_ region using DEMS on Pt in *n*-butanol containing acidic solution. The decrease with the increase in ω for the 2-butanol confirms that the CO_ads_ coverage cannot be the reason for a decrease in peak current as the formation of ketonic species is the dominant reaction at peak a_1_ for 2-butanol [60], which can easily diffuse away from the catalyst surface on rotation. In addition, peak a_1_ for 2-butanol is not affected by water oxidation as the potential of peak a_1_ is significantly lower compared to other primary alcohol molecules. Since diffusion of ketonic species reduces the further oxidation of ketone, the total current decreases. As no CO_ads_ but CO_2_ was observed during 2-butanol oxidation [4], the CO_2_ might have formed from butanone at high potential (Scheme 3b).

An initial increase in the current density was observed, up to 100 rpm, followed by a decrease with increasing ω for butanol oxidation (Fig. 2), similar to that observed for the ethanol oxidation reaction (Fig. 1). It could be presumed that the diffusion of soluble species from the electrode is not dominant at low ω compared with the transport of alcohol to the electrode under the experimental conditions employed. To further investigate whether the initial increase up to 100 rpm is due to the insignificant diffusion of intermediates, the rotation was increased directly to 900 from 0 rpm during 2-butanol oxidation (Fig. S3). A decrease in current was observed in this case indicating that at higher rotation the intermediates diffuse away whereas at 100 rpm this is not the case.

Peak a_2_ for *n*-butanol also showed a similar trend to that of ethanol, i.e. an initial increase and then a stable peak current with further increases in ω (Fig. S2). The peak a_2_ also showed a decrease in the peak current for 2-butanol (Fig. S2d) as in the case of methanol.

### Alcohol Oxidation Reactions on Carbon-Supported Pt Catalyst (Pt/C)

**Fig. 3:**
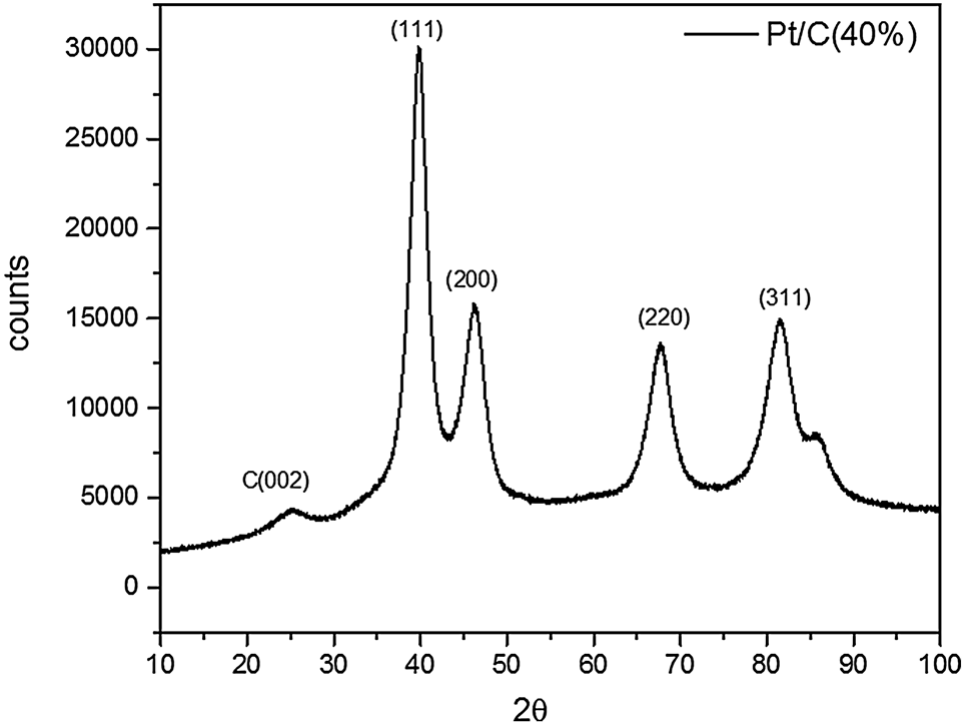
XRD spectra of the commercial Pt/C (40%) catalyst. The XRD peak shows the fcc structure of Pt particles

The XRD of Pt/C (40%) commercial catalyst is given in Fig. 3. A characteristic feature of face centred cubic (fcc) structure of Pt was obtained. The crystallite size (*d*) and lattice constant (*a*_*fcc*_) from Pt (220) plane was ca. 3.3 nm and 391.4 pm respectively.

#### Methanol and 2-Butanol Oxidation on Pt/C

**Fig. 4:**
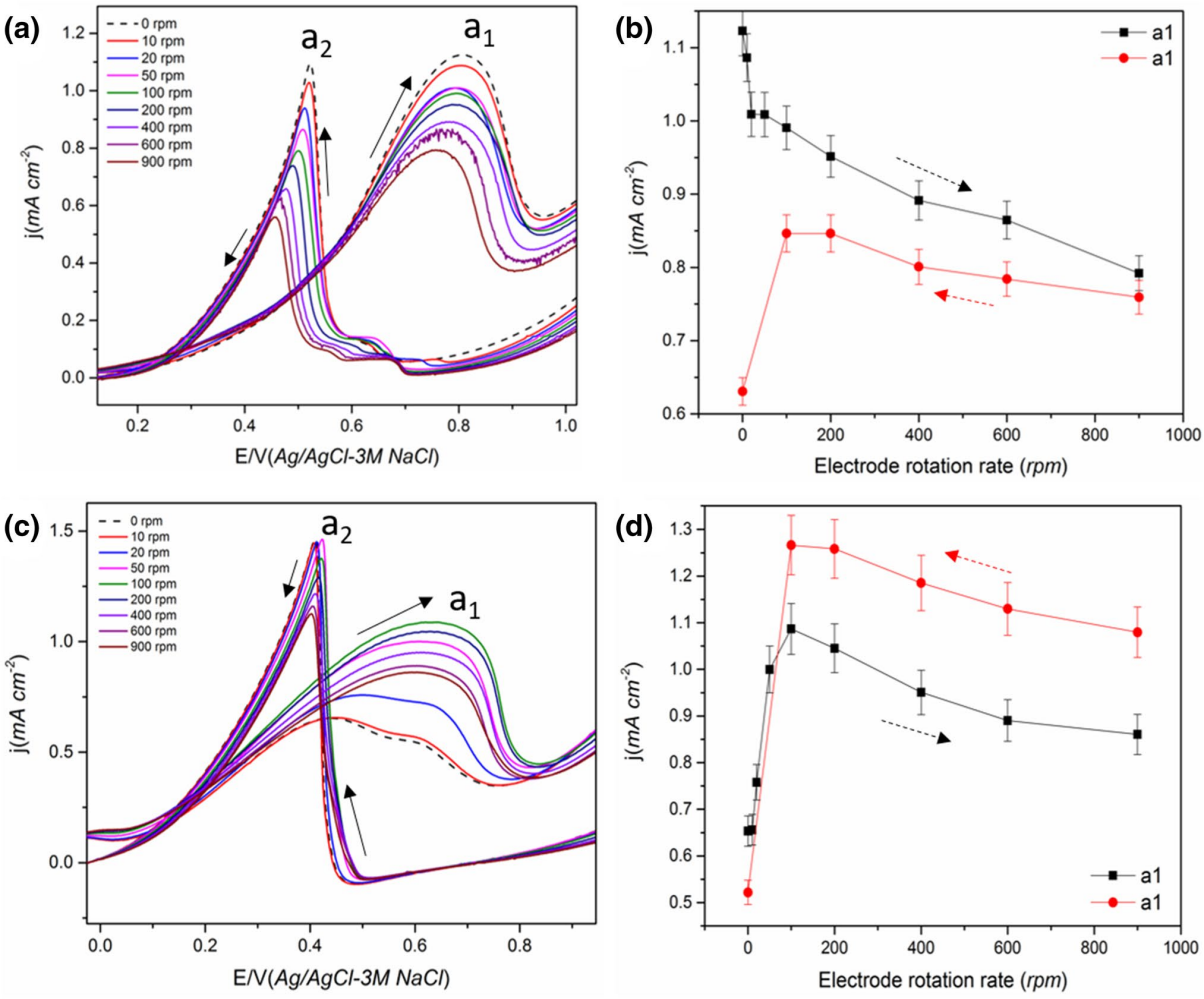
**a, b** The effect of electrode rotation on Pt/C (40 wt%) catalyst in 0.5 M methanol + 0.1 M HClO_4_ solution and evolution of peak a_1_ current. **c, d** The effect of electrode rotation on the peak current of Pt/C (40%) catalyst in 0.5 M 2-butanol + 0.1 M HClO_4_ solution. Scan rate 50 mVs^−1^ Solid arrow shows the direction of the potential scan

The effect of ω on carbon supported Pt nanoparticle (Pt/C) in methanol and 2-butanol containing solution is given in Fig. 4. Similar to the electrodeposited Pt film, a decrease in current with an increase in ω was observed for both alcohols. This indicates that the diffusion of soluble species could also be dominant on Pt/C. However, an increase in peak current with rotation for Pt/C has been reported for MOR when a higher catalyst loading of Pt/C was used [47]. Sayadi and Pickup [47] used a loading of 10 mg cm^−2^ of 20% Pt/C and observed an increase in methanol oxidation peak current on rotation of the electrode. The discrepancy with the current result could be due to the relatively lower catalyst loading used in the current study. When a thicker catalyst layer (0.14 mg Pt cm^−2^) was used, the peak current density did not decrease with rotation (Fig. 6). This confirms that at higher catalyst layer thickness (loading), the removal of the HCHO is restricted and the reaction becomes independent of the rotation rate. Gojkovic [42] also observed non-dependence of peak current with rotation for MOR on Pt/C catalyst. Sayadi and Pickup [51] observed a non-linear Koutecky–Levich (K–L) plot for EOR on Pt/C when thin catalyst layer was used indicating a lack of pure diffusion controlled reaction on the thin catalyst layer. At an even higher catalyst layer thickness, an increase in peak current with rotation rate could be expected as a complete oxidation of methanol to CO_2_ can occur leading to mass transport controlled reaction. In other words, it could be inferred that there is a threshold catalyst layer thickness for methanol oxidation (at a certain concentration and temperature) above which reaction become mass transport controlled and below which the reaction decreases. This threshold thickness value is different for different alcohol molecules as they have different reaction rates, reaction mechanisms and intermediate species formed. For example, in the case of ethanol at the same thickness as methanol (0.14 and 0.07 mg cm^−2^) the peak current decrease (Fig. 6). So the threshold catalyst loading for ethanol oxidation reaction is lower than that for methanol.

An increased CO_2_ efficiency on Pt/C (ca. 88% on 0.6 mg Pt cm^−2^) compared with the electrodeposited Pt film (ca. 30%) has been reported in DEMS studies for MOR [21] indicating complete oxidation on carbon supported Pt catalysts. Childers et al. [37] observed that over carbon supported Pt and PtRu, methanol tends to undergo complete oxidation at lower potential as the retention time of the partial oxidation product HCHO is higher in these cases which help its further oxidation to CO_2_. Electrodeposited Pt film showed higher HCHO formation compared to Pt/C. It is clear from Fig. 4 that the peak currents at all ω’s are higher than those in the quiescent solution, which, is in agreement with the higher retention time of intermediates on the catalyst surface and the improved overall oxidation efficiency as well as improved transport of alcohol to the catalyst surface.

#### Ethanol and *n*-Butanol Oxidation on Pt/C

**Fig. 5:**
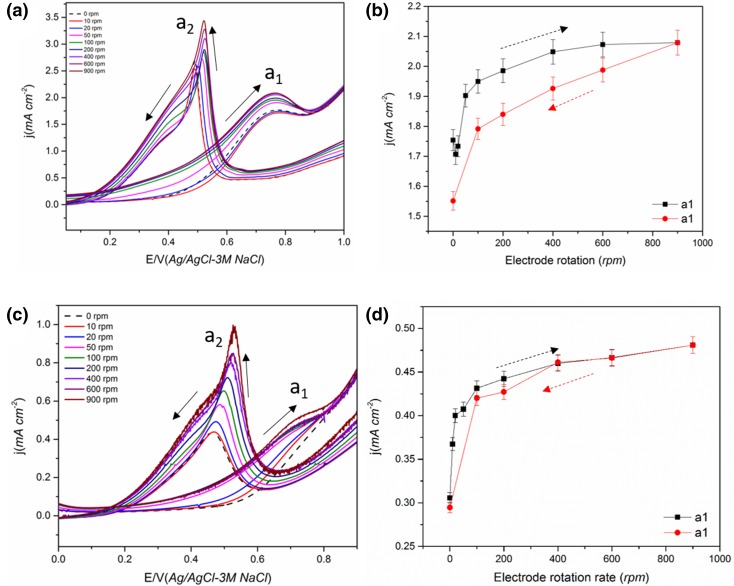
**a, b** The effect of electrode rotation in 0.5 M ethanol + 0.1 M HClO_4_ solution on Pt/C (40 wt%) catalyst and the evolution of peak a_1_ current during the increase and decrease in the ω. **c, d** The effect of electrode rotation in 0.5 M *n*-butanol + 0.1 M HClO_4_ on Pt/C (40 wt%) catalyst and the evolution of peak a_1_ current on increase and decrease in ω

**Scheme 4:**
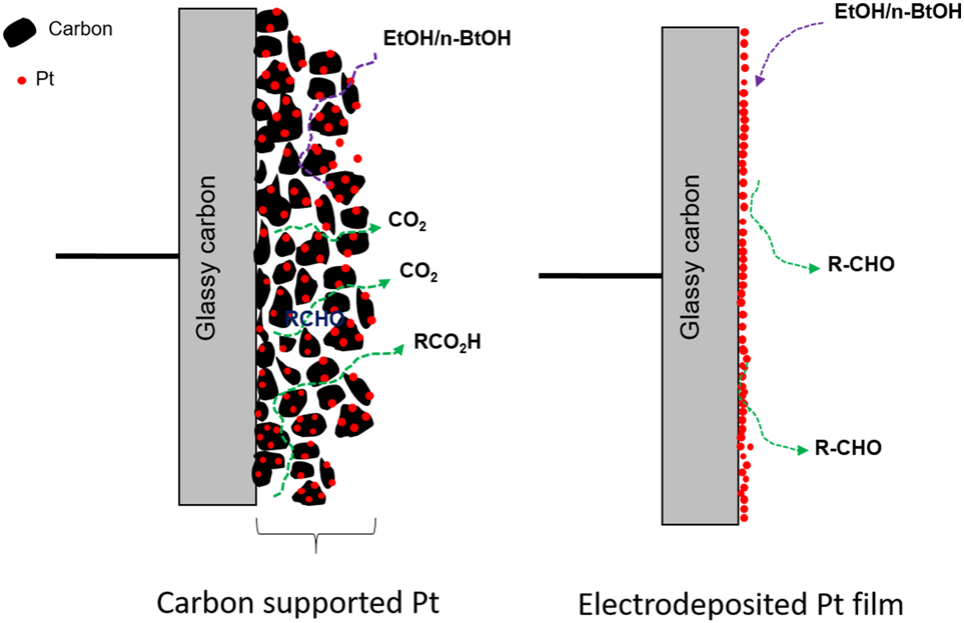
The schematic of the effect of rotation rate (ω) on carbon supported and electrodeposited Pt film electrode

In contrast, in the case of ethanol (Fig. 5a) and *n*-butanol (Fig. 5c), an increase in peak current was observed with increase in ω. A similar trend was also reported previously for ethanol oxidation on Pt/C for both peaks a_1_ and a_2_ [50, 51]. This was attributed to the enhancement of the fast ethanol supply to the active sites free of ethanol adsorbates which formerly oxidised into acetaldehyde (kinetics of this reaction is fast). A higher residence time of intermediate species on the carbon supported catalyst layer and subsequent hindrance of the diffusion of soluble species away from the corrugated Pt/C surface leading to a more complete oxidation [21, 42, 50]. Also, the intermediates formed on Pt/C may remain in the proximity of the catalyst surface increasing the chance of re-adsorption and further oxidation to CO_2_ (Scheme 4). It has to be noted the Pt metal loading for the Pt/C (0.07 mg cm^−2^) was ca. six times lower than that for the electrodeposited film (0.46 mg cm^−2^) and still an increase in current was observed indicating that rough carbon surface has a significant influence on the diffusion of reactive species. Katayanagi and Yamazaki [46] studied the effect of flow rate on the ethanol oxidation reaction on the anode of a single cell and observed that with an increase in flow rate, the CO_2_ production decreases. In addition, the CO_2_ production increases when acetaldehyde production decreases on the catalyst surface, i.e. with an increase in flow rate, more acetaldehyde is produced. Sayadi and Pickup [51] proposed that the increase in current could also be due to the fact that CH_3_CHO acts as a poison for the catalyst and on a thick catalyst layer the oxidation of CH_3_CHO to CH_3_COOH becomes easier. The removal of CH_3_CHO thus increases the current due to the improved adsorption of alcohol. Peak a_2_ was found to vary more with rotation compared to peak a_1_ for all the alcohols tested on Pt/C. A similar observation was also made by Sayadi and Pickup [51] on Pt/C during EOR.

The average number of electrons (*n*_*av*_) transferred in the EOR on Pt/C is calculated using Koutecky-Levich plot $\left( {{i^{ - 1}}{\text{ vs.}}{\omega ^{ - 1/2}}} \right)$ (Fig. S5) as per Eq. (8) [47, 51, 52] where ${i_k}$ is the kinetic current, ${i_L}$ is the limiting current (Levich current), *n*_*av*_ is the number of electrons transferred in the reaction, *A* is the area of the electrode, *F* is the Faraday constant (96,485 C mol^−1^), *D* is the diffusion coefficient of the alcohol molecule (1.22 × 10^−5^ cm^2^ s^−1^ for aqueous ethanol at 25 °C) [51], $~\nu$ is the kinematic viscosity (1.0 × 10^−2^ cm^2^ s^−1^) [47, 51], *C* is the concentration (mol cm^−3^) and ${\upomega}$ is the rotation rate (radians per second).

8 $$\frac{1}{i}=\frac{1}{{{i_k}}}+\frac{1}{{{i_L}}}=\frac{1}{{{i_k}}}+\frac{1}{{0.62{n_{av}}FA{D^{2/3}}{\nu ^{ - 1/6}}C{\omega ^{1/2}}}}$$

**Fig. 6:**
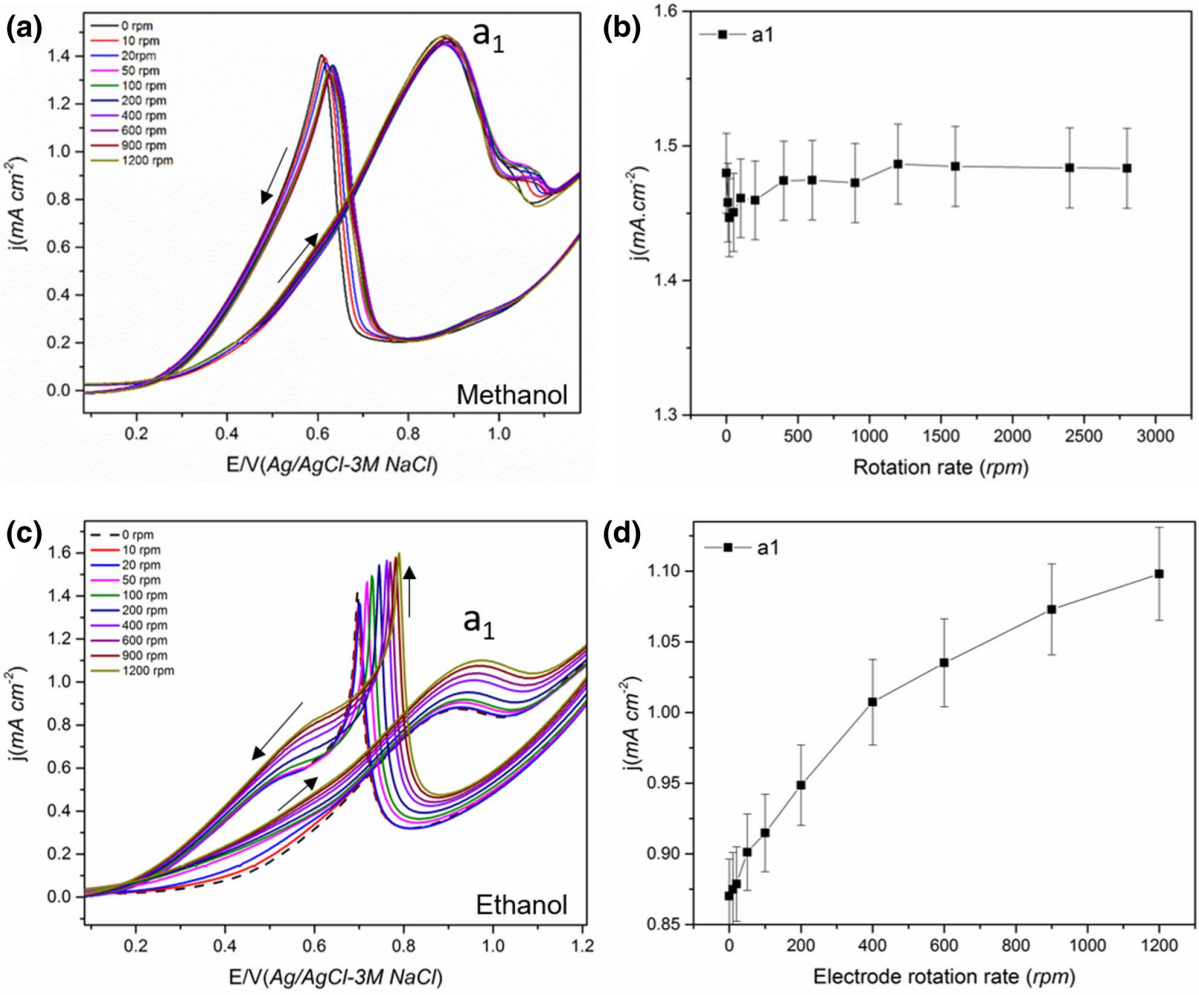
The effect of electrode rotation rate on the CV and peak a1 current of (**a, b**) 0.5 M methanol + 0.1 M HClO_4_ and (**c, d**) 0.5 M ethanol + 0.1 M HClO_4_ solution on a thicker Pt/C catalyst layer (0.14 mg cm^−2^)

Using a literature value of ethanol diffusion coefficient in water at 25 °C, 1.22 × 10^−5^ cm^2^ s^−1^ [51], the average number of electrons (*n*) at 0.75 V was calculated to be 0.2, which is not a realistic value whereas when lower D value was used (1.22 × 10^−7^ cm^2^ s^−1^), the *n* value was found to be ~ 4.2 which is more realistic value for a partial oxidation of ethanol to acetic acid. This discrepancy in the value of D with the literature value indicates that back diffusion occurs during the rotation of the electrode making the net diffusion coefficient lower (Table 2). Moreover, the limiting current (*i*_*L*_) calculated using Eq. (8) for ethanol oxidation on Pt/C at 900 rpm was ca. 0.78 A using the parameters described in Table 3 which is large compared to what was obtained from the experiment and can only be due to the error in the diffusion coefficient value [52]. Such discrepancies in D value were also reported previously for MOR [52].

**Table 2:** The n_av_ and diffusion coefficient values from K-L plot for ethanol oxidation on Pt/C

Potential	n_av_ for D = 1.22 × 10^−5^ cm^2^ s^−1^	n_av_ for D = 1.22 × 10^−7^ cm^2^ s^−1^	D calculated from theoretical n_av_ value = 4 (cm^2^ s^−1^)	D calculated from theoretical n_av_ value = 12 (cm^2^ s^−1^)
0.75 V	0.20	4.20	1.31 × 10^−7^	2.52 × 10^−8^

Data from Fig. 5a

**Table 3:** Limiting current (*i*_*L*_) calculation of Pt/C catalyst for ethanol oxidation reaction

Variable	Value
Faradays constant (*F*)	96,485 C mol^−1^
Geometrical area (*A*)	0.196 cm^2^
Diffusion coefficient (*D*)	1.22 × 10^−5^ cm^2^ s^−1^
Kinematic viscosity (*ν*)	0.01 cm^2^ s^−1^
Ethanol concentration (*C*)	0.5 mol cm^−3^
Electrons transferred (*n*)	12
ω at 900 rpm	94.2 rad s^−1^
Limiting current (*i*_*L*_)	0.78 A

The similarity between methanol and 2-butanol and that between ethanol and *n*-butanol, on both electrodeposited Pt film and Pt/C is noticeable. Methanol and 2-butanol are the most reactive alcohols whereas ethanol and *n*-butanol have relatively sluggish reaction kinetics. Less reactive molecules such as ethanol and *n*-butanol will not change the Pt active surface as significantly compared to more reactive molecules such as methanol and 2-butanol due to lower reaction intermediate products formed. A large number of intermediates (e.g. HCHO, CO_ads_) could be formed during MOR and a lower Pt/C loading might not be sufficient to hinder its diffusion away from the electrode. Peak a_2_ also showed a similar trend with increasing rotation as peak a_1_ for both ethanol and *n*-butanol (Fig. S4). This was confirmed by doubling the Pt/C catalyst loading (Fig. 6). In this case, the methanol oxidation peak current was independent of the rotation rate whereas, for ethanol, the peak current was found to be increasing with an increase in rotation rate as found for the lower catalyst loading case. As mentioned in Sect. 3.3.1, this could be due to the increased resident time of the MOR reaction intermediates in the catalyst. The threshold catalyst loading above which the reaction becomes mass transport limited is lower for ethanol than methanol due to the sluggish kinetics of EOR.

From the above results, it can be concluded that the increase in current on carbon supported catalyst is likely to be a combination of (i) a change in the reaction mechanism from a CO_ads_ dominant pathway on electrodeposited Pt film to being partial oxidation dominated on Pt/C, and/or, (ii) relatively poor removal of the intermediates and higher chances of re-adsorption of intermediates in presence of a carbon supported Pt surface. Hayes et al. [29] observed a decrease in peak current with an increase in the ω for Pt disk electrode in alkaline media whereas, for nanoporous Pt, the current was found to increase with an increase in ω. This was attributed to the trapping of non-adsorbed intermediate species inside the pores which otherwise would sweep away on rotation increasing the chance of its further oxidation. Zhang et al. [61] also observed a higher activity of a porous Au electrode for MOR and attributed this to the trapping of OH^−^ ion in the pores. These, along with the results obtained, herein, indicate the importance of increasing the residence time of the intermediates on the catalyst surface in designing new more efficient catalysts.

## Conclusions

The mass transport effects on the electro-oxidation of various alcohol molecules (methanol, ethanol, *n*-butanol and 2-butanol) on both electrodeposited Pt and carbon supported Pt (Pt/C) catalysts were studied systematically to understand the catalyst activity under non-steady state conditions. A clear dependence of the effect of mass transport on the thickness of the catalyst layer and the type of the alcohol molecules were observed where the electrodeposited film was used as the model system for the thinnest catalyst layer and carbon supported Pt was used as a model to thicker and corrugated catalyst surface. It was found that increasing mass transport has a negative effect on the oxidation of the alcohol molecules on thinner catalyst layer (electrodeposited Pt film). The decrease in peak current is attributed to a combination of factors; (i) removal of the partial oxidation products from the catalyst surface making it unavailable for further oxidation and/or (ii) a shift in the dominant reaction mechanism pathway to the one forming more strongly adsorbed intermediates under rotation condition, blocking the Pt active sites.

On the other hand, on the Pt/C catalyst surface two different trends were observed. On a thinner Pt/C catalyst layer (i.e. lower loading), the methanol and 2-butanol oxidation current decrease with increase in rotation. However, on a higher thickness of the catalyst layer, the decrease was prevented and a steady state current was maintained for MOR. A further increase in catalyst loading is expected to increase the current as in the case of ethanol (experiment not carried out). In the case of ethanol, for the same loading, the current was observed to increase with an increase in rotation. In other words, a threshold thickness/loading can be identified for each alcohol molecule above which the reaction becomes mass transport limited. This threshold thickness value is assumed to be higher for reactive alcohols compared to the less reactive alcohols. Highly reactive molecules (eg: methanol and 2-butanol) could form significant number of intermediates which may cover/block most of the Pt sites and change the Pt surface significantly. At the carbon supported catalyst, the diffusion of soluble products are hindered and the chances of re-adsorption are improved (Scheme 4) as the residence of time of reactive species on the corrugated surface of carbon is higher.

Since a DAFC usually operates on continuous fuel supply mode, not all catalyst particles on the electrode surface may get a uniform fuel concentration and thus the dependence of activity on mass transport has to be considered when designing and optimising the electrode structures [41]. It is significant from the data obtained for Pt/C systems that the decrease in activity could be compensated by a suitable catalyst design to increase the resident time of the intermediates such as porous carbon supported catalyst and/or porous catalyst structure. Further studies are required to identify the interaction between the intermediates and support, the effect of porous morphology vs. retention time of intermediates etc. to gain more insight into the activity and to design new more effective catalysts for alcohol electro-oxidation reactions.

## Electronic Supplementary material

Below is the link to the electronic supplementary material.

Supplementary material 1 (TIF 118 KB)

Supplementary material 2 (TIF 414 KB)

Supplementary material 3 (TIF 74 KB)

Supplementary material 4 (TIF 430 KB)

Supplementary material 5 (PDF 1200 KB)
