# Supplementary figures and images for: Effect of Mass Transport on the Electrochemical Oxidation of Alcohols Over Electrodeposited Film and Carbon-Supported Pt Electrodes
Source: Top Catal. 2018 Jan 19;61(3):240–53. doi: 10.1007/s11244-018-0893-6 (PMC6413813; doi:10.1007/s11244-018-0893-6)

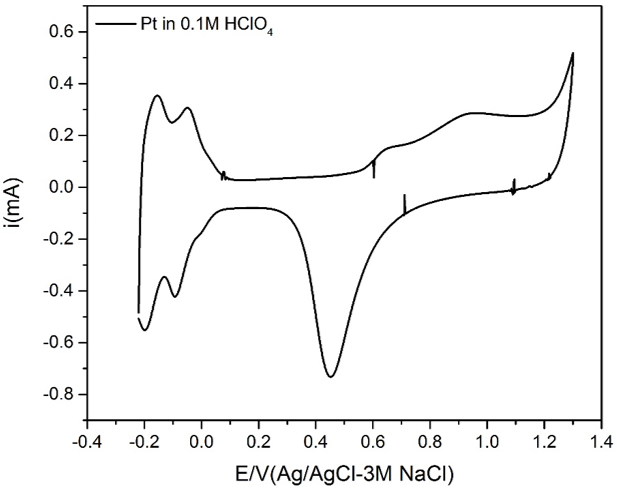

Supplement: Supplementary file 1 — Supplementary material 1 (TIF 118 KB) [file 11244_2018_893_MOESM1_ESM.tif]

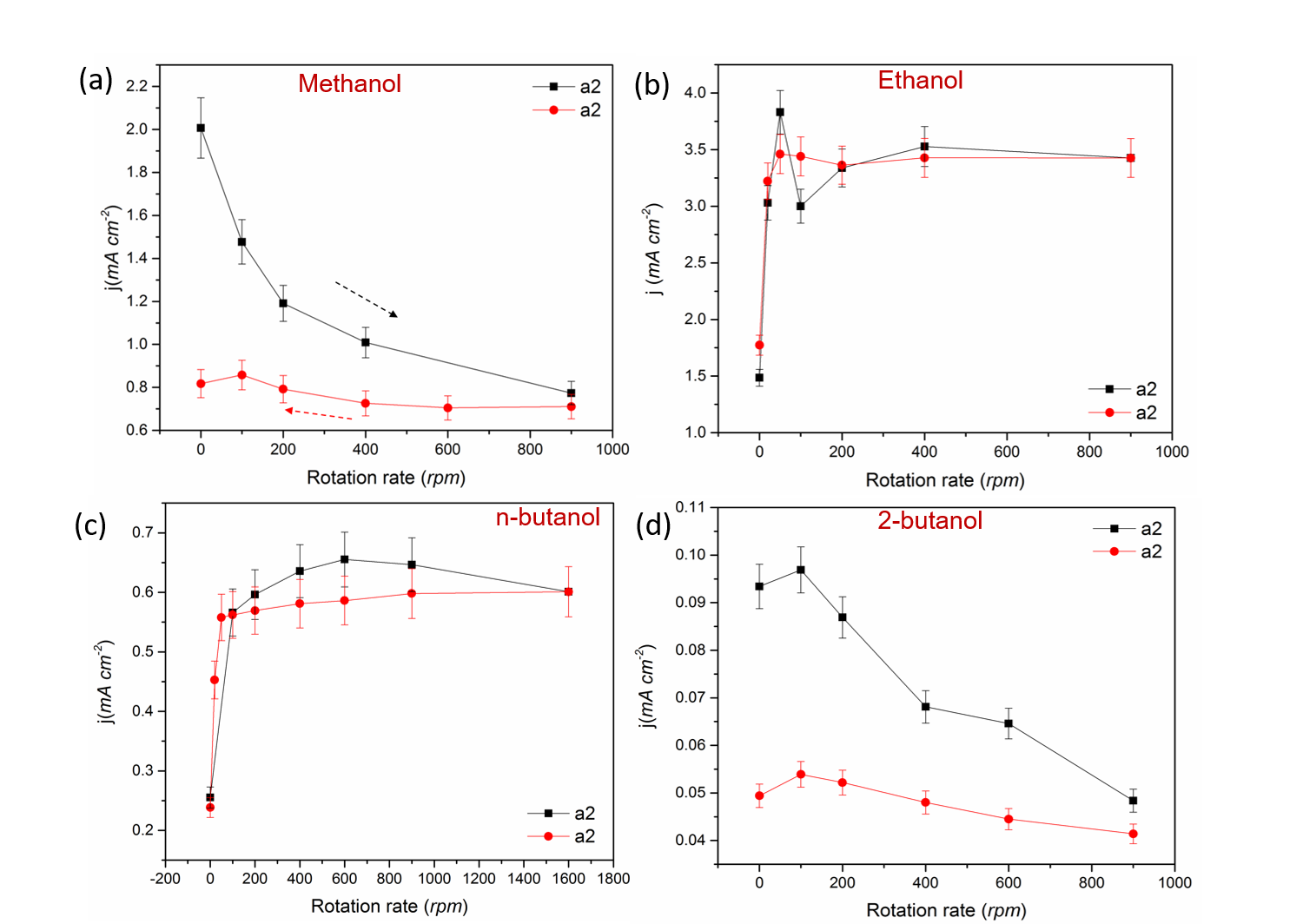

Supplement: Supplementary file 2 — Supplementary material 2 (TIF 414 KB) [file 11244_2018_893_MOESM2_ESM.tif]

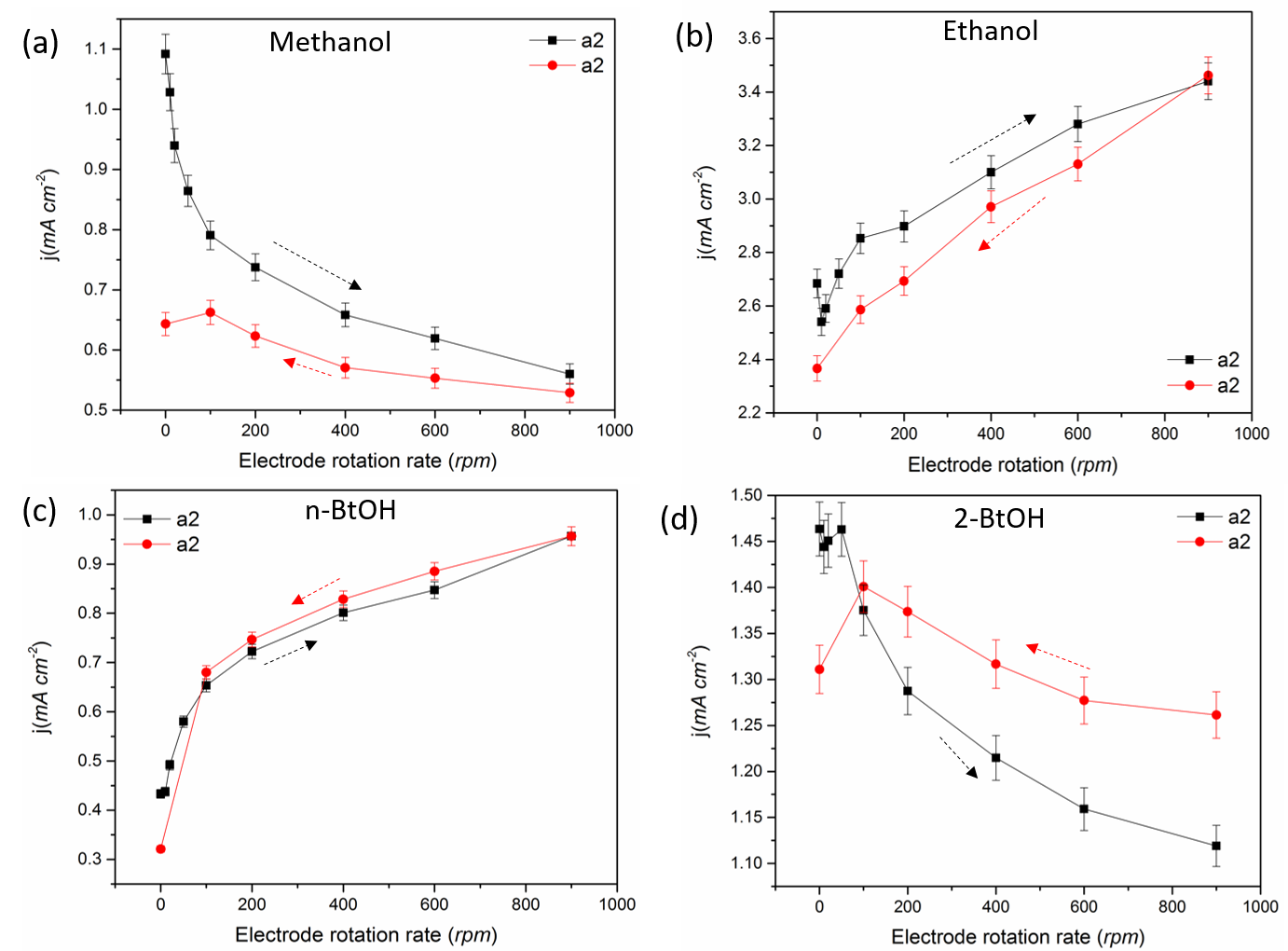

Supplement: Supplementary file 4 — Supplementary material 4 (TIF 430 KB) [file 11244_2018_893_MOESM4_ESM.tif]
