# Supplementary material for: Effect of Mass Transport on the Electrochemical Oxidation of Alcohols Over Electrodeposited Film and Carbon-Supported Pt Electrodes
Source: Top Catal. 2018 Jan 19;61(3):240–53. doi: 10.1007/s11244-018-0893-6 (PMC6413813; doi:10.1007/s11244-018-0893-6)
Supplement: Supplementary file 5 — Supplementary material 5 (PDF 1200 KB) [file 11244_2018_893_MOESM5_ESM.pdf]

Supplementary Information

Effect of Mass Transport on the Electrochemical Oxidation of Alcohols over Electrodeposited film and Carbon- Supported Pt electrodes

Vinod Kumar Puthiyapura[1], Wen-Feng Lin*[,2], Andrea E Russell[3], Dan J L Brett[4], Christopher Hardacre*[,1]

[1]School of Chemical Engineering and Analytical Science, The University of Manchester, Manchester-M13 9PL, UK.
[2]Department of Chemical Engineering, Loughborough University, Loughborough, Leicestershire-LE1 13TU, UK

[3]Department of Chemistry, University of Southampton, High field, Southampton- SO17 1BJ, UK
[4]Department of Chemical Engineering, University College London (UCL), London- WC1E 7JE, UK.

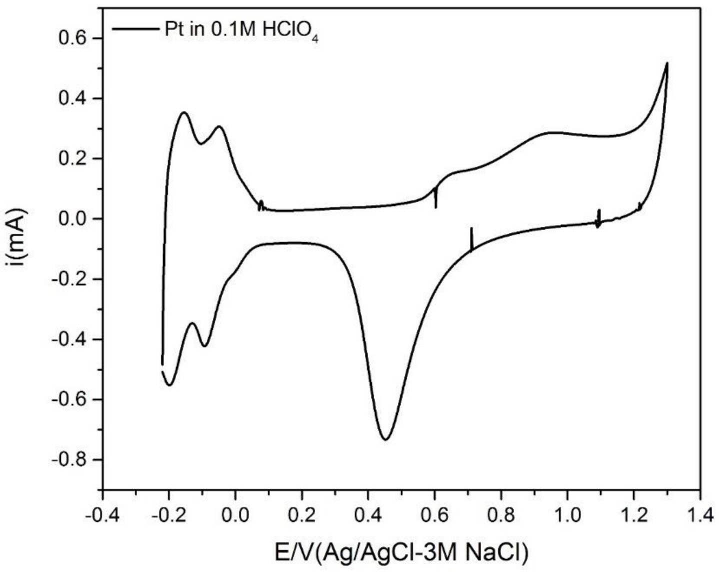

Figure S1. A typical voltammogram of electrodeposited Pt in 0.1M $HClO_4$ solution. Scan rate 50 mVs^{-1}. Pt active area (A_r) was calculated from the H_{UPD} desorption region.

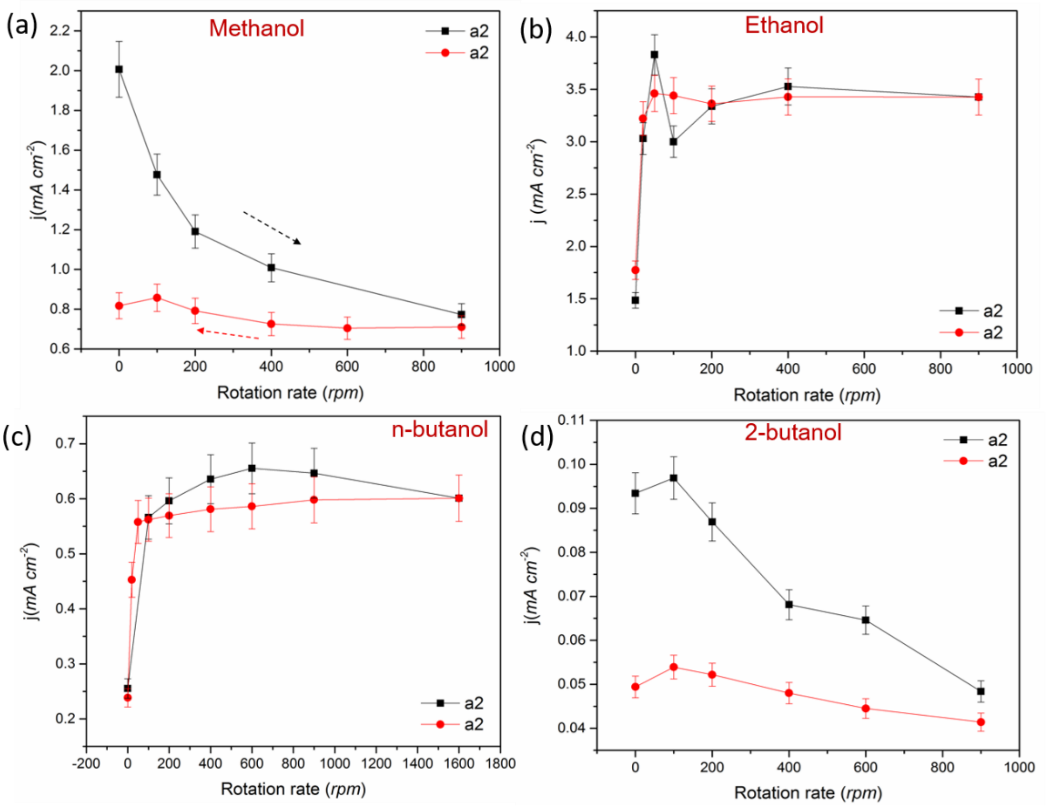

Figure S2. The effect of electrode rotation on peak **a_2** current for electrodeposited Pt film in solutions of (a) 0.5M methanol+0.1M $HClO_4$ (b) 0.5M ethanol+0.1M $HClO_4$ (c) 0.5M *n*-butanol+0.1M $HClO_4$ (d) 0.1M 2-butanol+0.1M $HClO_4$. Scan rate 50 mVs^{-1}.

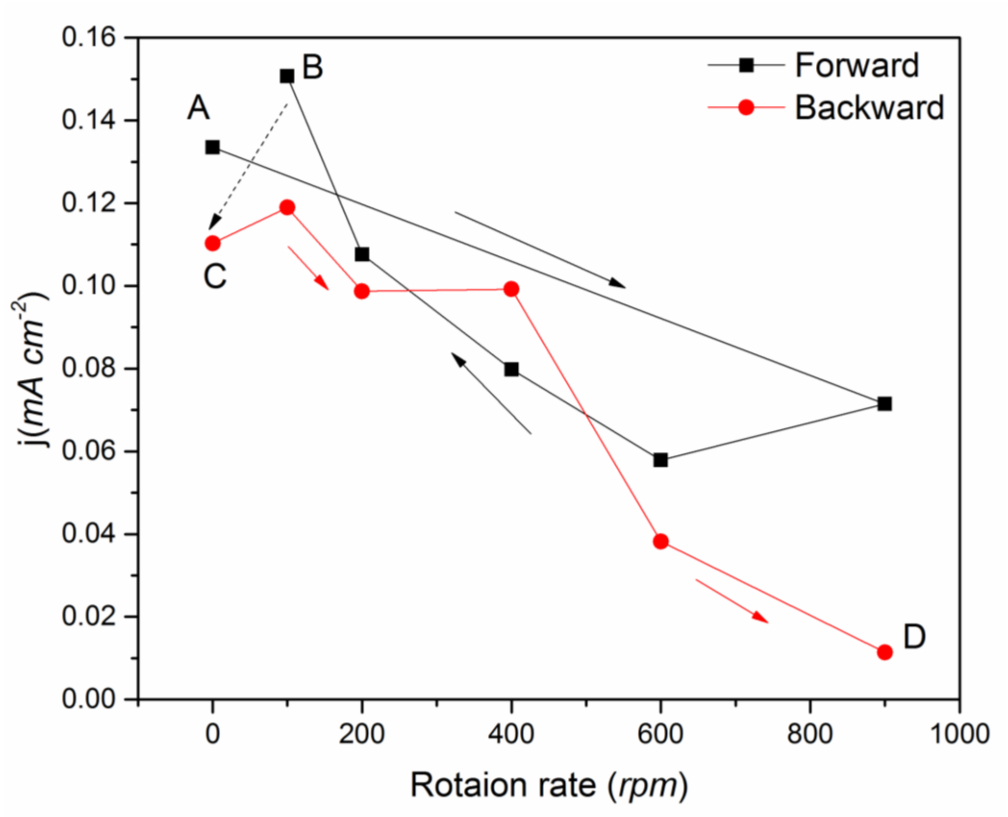

Figure S3. The effect of electrode rotation rate on peak **a_1** current density of electrodeposited Pt film on 0.1 M 2-butanol+0.1 M $HClO_4$ where electrode rotation changed from 0 rpm to 900 rpm directly. The rotation rate changed from A to B (increase & then decrease in ω) and C to D (increase in ω) as given in the figure.

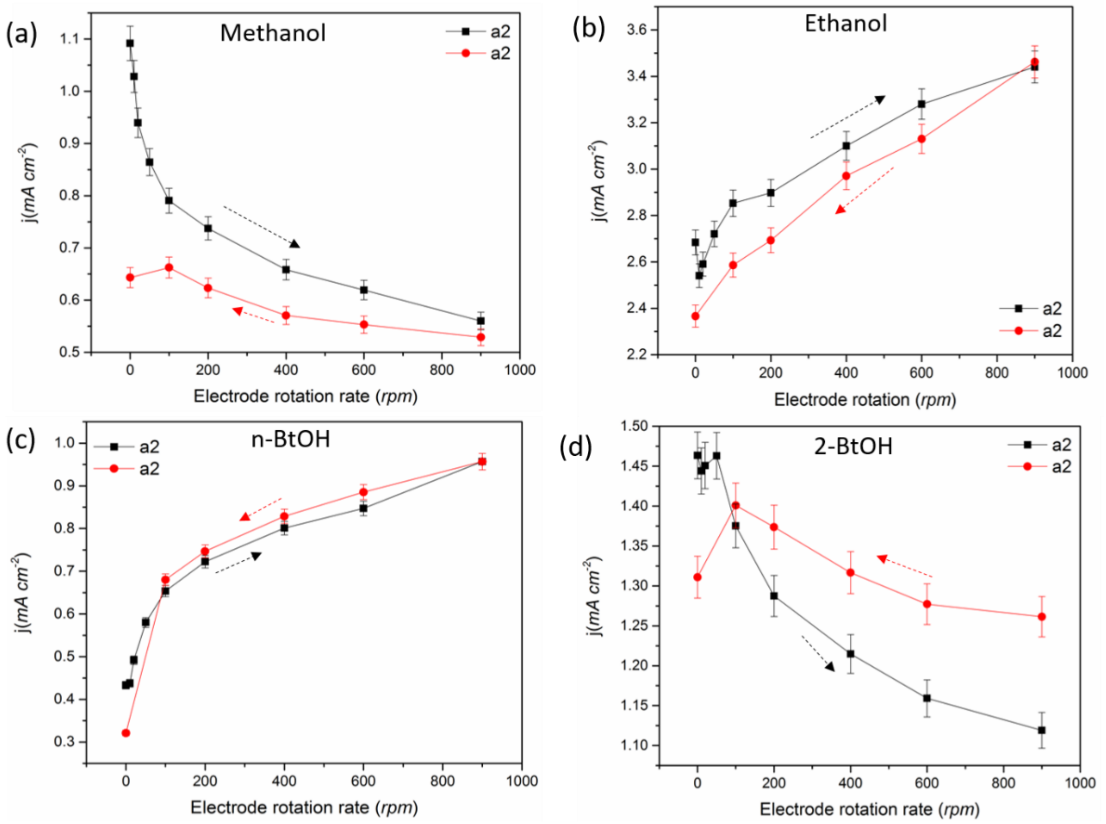

Figure S4. The effect of electrode rotation rate on peak **a_2** current density for Pt/C(40%) catalyst in 0.5M alcohol +0.1M $HClO_4$ solution of (a) methanol (b) ethanol (c) *n*-butanol(d) 2-butanol. Scan rate 50 mVs^{-1}.

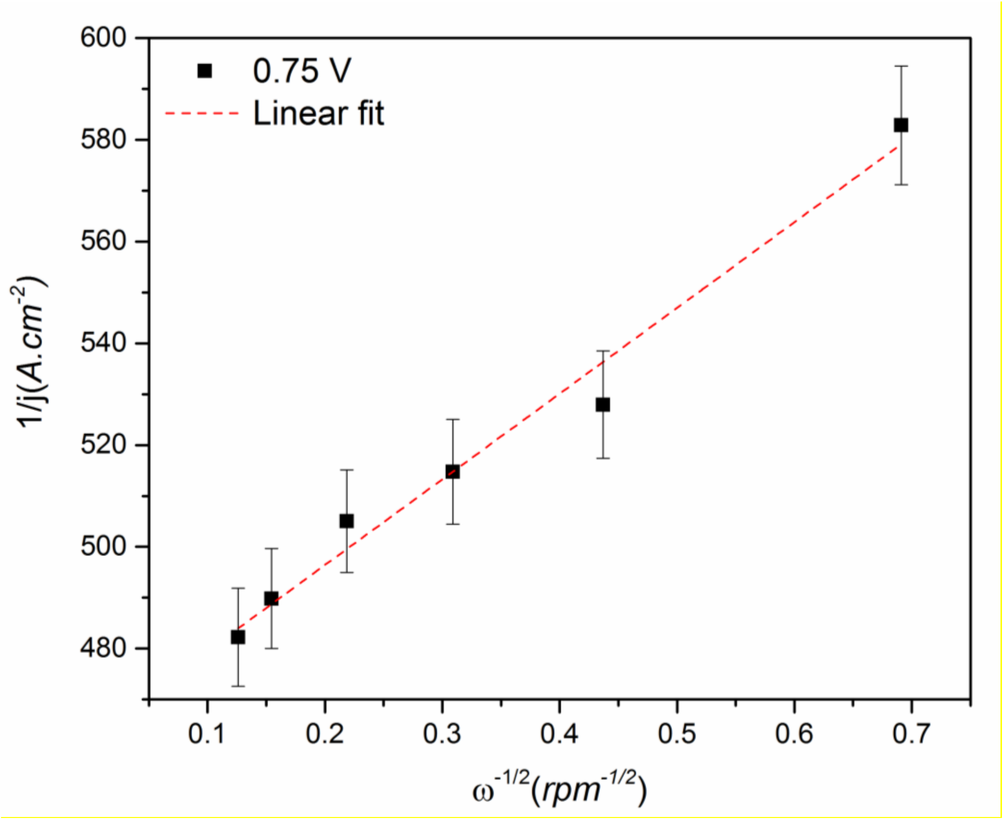

Figure S5. Koutecky-Levich plot of ethanol oxidation reaction on Pt/C at 0.75 V.
